# Enclosed Electronic System for Force Measurements in Knee Implants

**DOI:** 10.3390/s140815009

**Published:** 2014-08-14

**Authors:** David Forchelet, Matteo Simoncini, Arash Arami, Arnaud Bertsch, Eric Meurville, Kamiar Aminian, Peter Ryser, Philippe Renaud

**Affiliations:** 1 Microsystems Laboratory (LMIS4), École Polytechnique Fédérale de Lausanne (EPFL), CH-1015 Lausanne, Switzerland; E-Mails: arnaud.bertsch@epfl.ch (A.B.); philippe.renaud@epfl.ch (P.R.); 2 Laboratory of Microengineering for Manufacturing (LPM), École Polytechnique Fédérale de Lausanne (EPFL), CH-1015 Lausanne, Switzerland; E-Mails: matteo.simoncini@epfl.ch (M.S.); eric.meurville@epfl.ch (E.M.); peter.ryser@epfl.ch (P.R.); 3 Laboratory of Movement Analysis and Measurement (LMAM), École Polytechnique Fédérale de Lausanne (EPFL), CH-1015 Lausanne, Switzerland; E-Mails: arash.arami@epfl.ch (A.A.); kamiar.aminian@epfl.ch (K.A.)

**Keywords:** total joint arthroplasty, strain sensor, load monitoring, instrumented implant, biomechanics

## Abstract

Total knee arthroplasty is a widely performed surgical technique. Soft tissue force balancing during the operation relies strongly on the experience of the surgeon in equilibrating tension in the collateral ligaments. Little information on the forces in the implanted prosthesis is available during surgery and post-operative treatment. This paper presents the design, fabrication and testing of an instrumented insert performing force measurements in a knee prosthesis. The insert contains a closed structure composed of printed circuit boards and incorporates a microfabricated polyimide thin-film piezoresistive strain sensor for each condylar compartment. The sensor is tested in a mechanical knee simulator that mimics *in-vivo* conditions. For characterization purposes, static and dynamic load patterns are applied to the instrumented insert. Results show that the sensors are able to measure forces up to 1.5 times body weight with a sensitivity fitting the requirements for the proposed use. Dynamic testing of the insert shows a good tracking of slow and fast changing forces in the knee prosthesis by the sensors.

## 1. Introduction

**Figure 1.**
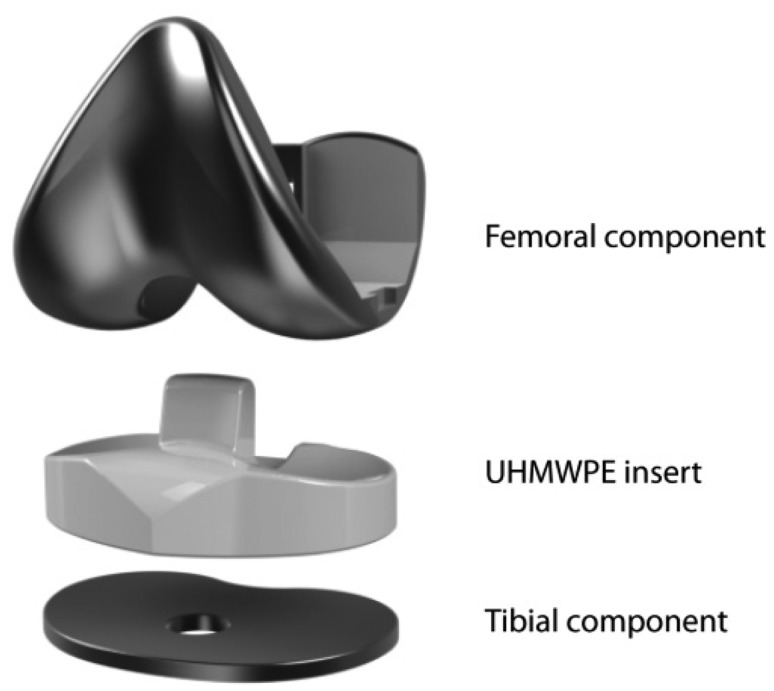
Total condylar prosthesis.

Knee osteoarthritis (OA) is one of the main causes of knee pain and disability in elderly population and its prevalence has been sharply increased in the recent years in developing countries [1]. Obesity, any history of knee injuries and extreme physical activities [2], combined with age factors contribute to the appearance of OA. To restore function in arthritic knees and alleviate crippling pain, knee replacements or total knee arthroplasty (TKA) surgeries have become an effective solution. With the seminal work of Insall *et al.* in the early 70s, a new concept for TKA prosthesis was introduced: total condylar prosthesis. Previously encountered problems of hinged prosthesis have been solved using this semiconstrained design that recreates both the patellofemoral and femorotibial joints. The total condylar prosthesis, as represented in Figure 1, is composed of three main parts for recreating the tibiofemoral joint: a femoral component, an ultra-high molecular weight polyethylene (UHMWPE) insert and a tibial component. Additionally, on the knee cap, a patella button can be assembled, which slides on the anterior part of the femoral component.

Although the design of such prostheses has seen some minor improvements, their functional principle is similar to that of the one created by Insall *et al.* TKA has now become a common operation, with over 600,000 surgeries per year in the United States alone [3] and it is the most efficient tool for solving OA-related knee pain. The success of this surgery has led to more and ever younger patient implantations, hence a need for improving stability and longevity of the implant and reducing the number of heavy revision surgeries.

Smart or instrumented prosthesis contain sensors that enable monitoring the position or mechanical state of the prosthesis through some telemetric method. Research efforts have been focused on using different technologies for force sensing such as strain gauges or Bragg gratings [4]. Instrumented implants typically rely on modifying the shape and functionality of the prosthetic parts, affecting surgical protocols and prosthesis performance thereby reducing the acceptance of the device amongst surgeons and patients.

Most instrumented knee prosthesis prototypes alter the metallic tibial component of the prosthesis [5–10]. However those devices involved thickening and extending the tibial part, leading to more bone removal during surgery. Additionally, they showed low remote powering efficiency [10].

The integration of the sensors and electronics in the polymer inserts [11–15] allows for wireless communication and a better remote powering efficiency [14] without adding any component or changing the external surface or function of the insert. However, the insert is a place of high strain and potential wear where the sensing system could potentially increase risk of failure. Such prosthesis malfunction or aging should be detected by monitoring the insert mechanical behavior.

Intra-operative devices based on insert instrumentation were designed for force measurement in the condyles [16]. Such tools, *i.e.*, Verasense^TM^ (Orthosensor, Diana Beach, FL, USA), have been commercialized, however they cannot be used for long term implantation. They incorporate a variety of sensors by modifying the insert material, mechanical behavior and shape, thereby limiting them to intra-operative use. The device presented in this paper has intra-operative and post-operative potential and could assist both with the surgical procedure and diagnostics of post-operative pain or discomfort. Data collected through the use of such a device could yield essential and previously unavailable information to prosthesis manufacturers.

When force sensors are assembled in the insert, whether magnetic [11] or piezoresistive [12], strong nonlinearities are observed due to the viscoelastic behavior of the polyethylene. Sensors assembled directly onto the UHMWPE are typically subject to creep and hysteresis. The sensor described in [12] uses similar technology to the one presented here and is subject to a high creep behavior that limits its usability. This particular sensor measured the transverse strain due to force in the condyle and is directly glued in a cut insert.

In previous work the validation of the force sensors was performed in an experiment using single or multiple axial servo-hydraulic load units [5,8,9,11,12]. Such systems do not recreate a realistic simulation of how the forces apply in a knee implant. In this work we perform validation of the force sensor in a robotic knee simulator [17]. In this simulator, we can apply different forces in different knee flexion angles and replicate gait force patterns previously measured on patients [18]. This testing setup fits more closely the condition encountered in *in-vivo* knee prosthesis implantation.

In this study, we fabricate and test a device for measuring force in each condylar compartment of a UHMWPE knee prosthesis insert. We propose to incorporate a thin film microfabricated strain sensor in a hollowed stack of FR4 PCB layers: measuring the deflection of this harder and low-creep material. This structure overcomes the viscoelastic limitations previously encountered and creates a versatile space for potential integration of remote powering and wireless communication units or vibration and kinematics sensors [19]. The device is an interesting surgical and diagnostic tool that could improve surgery quality and provide follow-up information. Furthermore, this implementation leads to a surgically non-disruptive insert in which the surface and behavior are not modified, hence potentially fostering its acceptance.

## 2. Sensor Design, Fabrication and Packaging

The device presented here integrates two force sensing gauges to measure forces separately in each condyle compartment of a total condylar prosthesis. The strain gauges are placed 15.5 mm away from the center of the intercondylar fossa under the condyle. They are positioned under the contact points between the femoral part and the insert.

**Figure 2.**
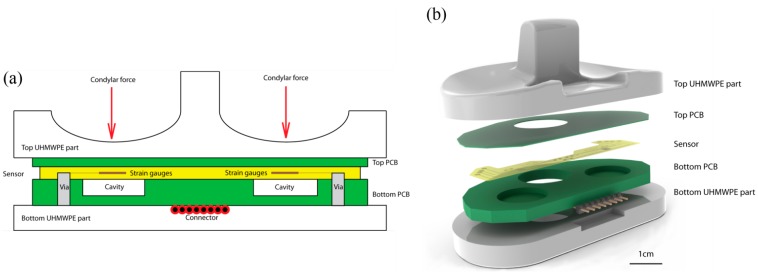
(**a**) Cross-section diagram of the instrumented prosthetics (**b**) Components of the instrumented prosthetics.

The device is composed of a sealed capsule incorporated in the polyethylene insert of the knee prosthetics. The capsule is composed of two bonded layers of FR4 (support material for Printed Circuit Boards (PCBs)): a 300 μm top layer and a hollowed 3 mm bottom layer. The bottom thicker layer contains two cavities, placed under each condyle, into which the thinner layer deflects. A thin film strain sensor is assembled to the thinner deflecting structure, and its output signal depends directly on the force applied on the condyle. The sensor is glued to the low-creep hard FR4 layer and measures its stretching and flexion. The capsule is designed to have all components at a 3 mm distance to all external surfaces to allow a clear barrier between the device and the body-prosthesis interface. In this study, the capsule containing the sensors and electronics is glued in between the UHMWPE sections of 5 mm minimal thickness. The additional thickness allows for wearing in those areas without exposing the capsule to bodily fluids. A schematic is shown in Figure 2.

**Figure 3.**
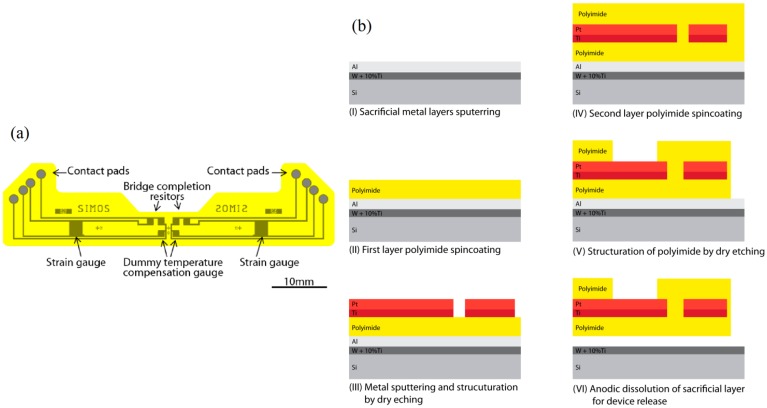
(**a**) Microstructured polymer thin film strain sensor. (**b**) Process flow for its microfabrication.

The strain gauge sensor is composed of a polyimide-metal-polyimide structure. The polyimide (PI) basis ensures electrical isolation and flexible mechanical support for the sensor while being biocompatible and highly chemically and mechanically stable [20]. The piezoresistive material is a platinum layer embedded in the polyimide layers: it is structured into 3.2 kΩ strain gauges and resistors as seen in Figure 3a. The temperature effect on the resistance of the gauge and expansion of the proof body are compensated for by addition of a dummy temperature compensation gauge for each strain gauge. For each condyle, a full Wheatstone bridge configuration is built into the sensor to avoid the need for external bridge completion elements. The dummy temperature compensation gauges and Wheatstone completion resistors are placed in an unstrained region, outside of the cavities, near the symmetry plane of the insert.

Micromechanical systems (MEMS) fabrication processes, summarized in Figure 3b, are used for the creation of the sensor. Figure 3 shows the fabrication process flow of the device in cross section. At first, a 70 nm layer of alloy WTi (10%) and a 1 μm Al sacrificial layer are sputtered onto a 525 μm thick <100> silicon wafer. A 5 μm layer of PI (PI2611, HD Microsystems, Hitachi, Tokyo, Japan) is spincoated onto the bare aluminum and cured at 300 °C for 2 h under a nitrogen flow. An adhesion promoting layer of 20 nm of Ti and a layer of 180 nm of Pt are sputtered onto the PI film. A 4 μm layer of photoresist (AZ1512, AZ Electronic Materials, Luxembourg) is photostructured and used for masking during dry reactive ion etching in Cl_2_ chemistry of the strain gauges and metal lines. A second PI layer of 5 μm is spun and cured in the same fashion as the first one. A 500 nm SiO_2_ layer is sputtered and a structured layer of AZ1512 is used as etch mask during dry etching in CF_4_. The PI is dry etched in O_2_ to create the contact pads and outline of the devices using the SiO_2_ structures as hard etch mask. The remaining SiO_2_ is removed through dry etching in CF_4_. The devices are liberated by anodic dissolution of the underlying Al layer by applying a 0.7 V bias between the Al and a Pt counter electrode in a salt saturated electrolyte as described in [21].

**Figure 4.**
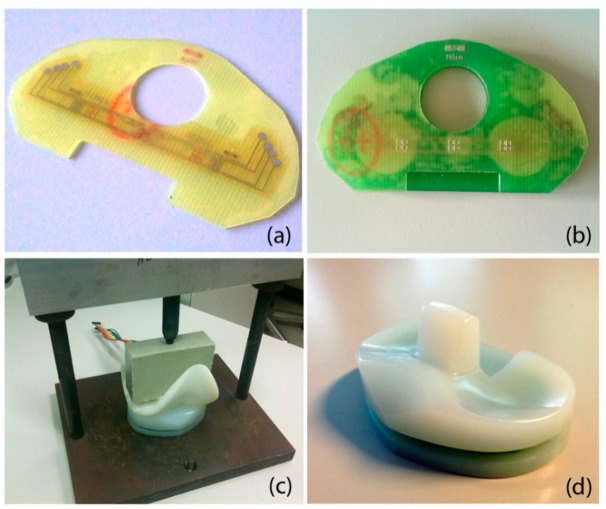
Microstructured polymer thin film strain sensor (**a**) thin film sensor glued on the thin top FR4 layer (**b**) complete capsule with sensor and PCB layers (**c**) gluing of the capsule in the UHMWPE section with applied pressure (**d**) resulting instrumented prosthesis.

Packaging of the sensor is done in a capsule of bonded FR4 layers and is shown in Figure 4. The top layer is composed of a 300 μm thick FR4 structure that acts as a deflecting membrane. After plasma cleaning, the thin film sensor is glued on the FR4 layer using biocompatible epoxy glue (301-2fl, Epotek, Billerica, MA, USA). Curing of the glue is performed in a press at 45 °C for 16 h. The thicker hollowed 3 mm PCB is assembled in a similar fashion. Conductive epoxy glue (H20E, Epotek) allows the contacting of the sensor's pad through the vias available in the bottom PCB. The conductive glue is cured for 16 h at 60 °C. An 8 pins male to male connector is soldered onto the bottom PCB for connecting the four points of the sensor's Wheatstone bridges. The capsule is assembled in the thinned top and bottom UHMWPE sections of an original insert, and glued using biocompatible flexible epoxy glue. Curing is again performed for 16 h at 45 °C under the pressure applied through a 3D printed femoral part mounted in a press.

This instrumented knee insert should conserve the mechanical and surface properties of the original insert. However, the use of a connector and the lateral exposed capsule prevent any *in-vivo* experiment. The integration of the electronic components needed for wireless communication in the thick layer as well as the embedding of the capsule in the insert during fabrication would potentially render the device implantable.

## 3. Experimental Setup and Results

**Figure 5.**
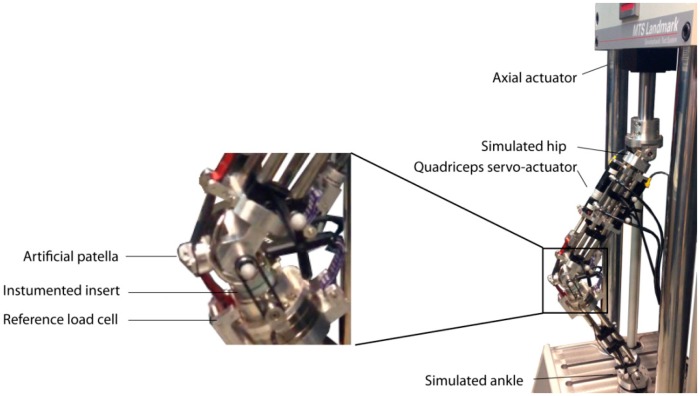
Robotic knee simulator.

To validate the fabricated instrumented insert, we fixed it in the robotic knee simulator. This robotic knee consists of an artificial femur and tibia which holds a prosthetic knee (see Figure 5). Multiple servo-hydraulic actuators, (MTS Bionix servo-hydraulic test system, MTS, Eden Prairie, MN, USA and Custom Actuators, Plymout, MN, USA) were used to mimic the muscles such as quadriceps (frontal muscle group on thigh) and hamstring (group of posterior thigh muscles) acting on the artificial knee [17]. In the current study, we used the frontal actuator which linked to an artificial patella (see Figure 5), in a force-control mode. A PID controller tuned for this actuator to track different patterns of reference forces such as amplitude modulated pulses, steps, and realistic patterns of knee forces measured in previous studies on smart knee prostheses [18]. In the experiments shown here, the sensor outputs are characterized against the force component perpendicular to the tibial plate measured by a reference load cell (ATI Industrial Automation, Apex, NC, USA) integrated in the artificial proximal tibia (see Figure 5).

The sensor electronic setup is a Wheatstone bridge in which all elements are structured into the thin-film sensor: both the resistor for temperature compensation and leg completion are embedded in the film. The contacts corresponding to the four vertices of the bridge are connected to a signal conditioning unit (SC-2345, National Instruments, Austin, TX, USA) through a full bridge module (SCC-SG04, National Instruments). The bridge is supplied with 2.5 V through the signal conditioning unit. The signal is fed into a data acquisition board (NI-Daqpad-6015, National Instruments) and recorded at a 100 Hz rate on a computer in a LabView environment.

**Figure 6.**
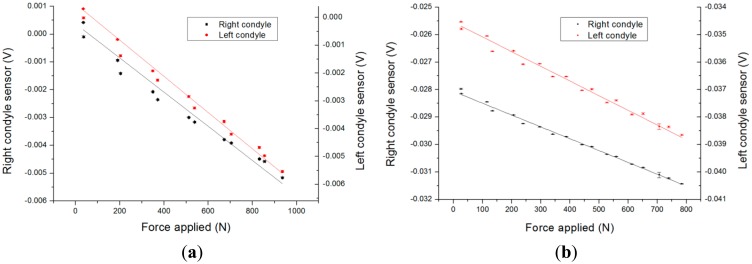
(**a**) Calibration curve at 10° knee flexion. For the right condyle sensor, sensitivity is determined to be 6.1 μV/N. For the left condyle sensor, sensitivity is determined to be 6.5 μV/N). (**b**) Calibration curve at 60° knee flexion. For the right condyle sensor, sensitivity is determined to be 4.3 μV/N. For the left condyle sensor, sensitivity is determined to be 5.3 μV/N.

Figure 6 represents the output voltages of the sensor channels during a loading experiment using a quadriceps actuation. The sensor exhibits a linear behavior against the total force measured perpendicularly to the tibial plate of the prosthetic implant upon application of step forces ramping from 0 to 900 N and back down.

A linear regression on this data yields sensitivities of 6.1 μV/N and 4.3 μV/N for the right condyle sensor at 10° and 60° knee flexion respectively. Similarly, it results in 6.5 μV/N and 5.3 μV/N for the left condyle sensor corresponding 10° and 60° knee flexion respectively. The statistics on the obtained calibration curves are provided in Table 1. The errors are defined here as deviations from the linear fit curve. The observed sensitivity decreases when the knee flexion angle increases. This phenomenon is due to a change in contact point between the femoral part and the polyethylene insert. If the contact point moves away from the center of the cavity, the flexion of the thinned PCB layer diminishes: this explains the sensitivity changes of this system associated to a change of contact point.

**Figure 7.**
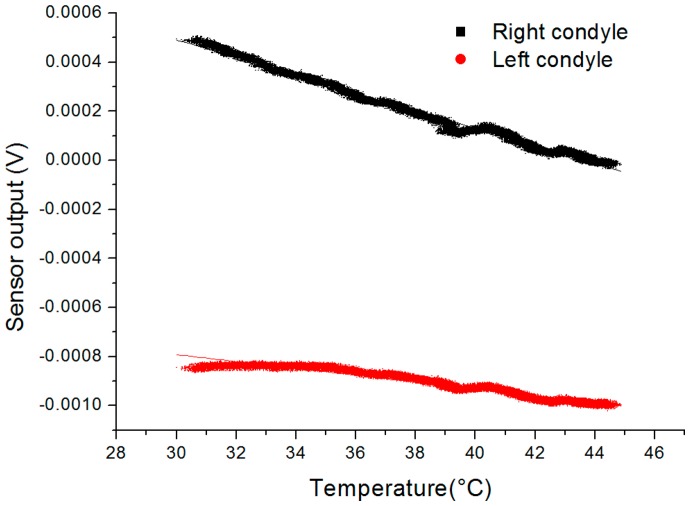
Calibration in temperature of the sensor. Sensitivity to temperature is determined to be 35.1 μV/°C for the right sensor and 13.9 μV/°C for the left sensor.

In addition to the sensitivity of the device in flexion angle, its sensitivity in temperature was investigated and reported in Figure 7. The equipment used for this experiment is composed of the instrumented insert and a reference thermocouple placed in a metal box to homogenously heat the devices and reduce conductive and convective cooling in air. The box is heated with a hot plate while the output voltage and temperature are continuously recorded using the previously described acquisition setup. The calibration was performed for temperature ranging from 30 to 45 °C and the linear fitting of the obtained output yields the following sensitivities: the right condyle sensor has a sensitivity of 35.1 μV/°C (0.98 adjusted-R^2^) and the left condyle sensor has a sensitivity of 13.9 μV/°C (0.94 adjusted-R^2^). Even though dummy temperature compensation resistors were included in the device, the dilating complex mechanical structure and slight mismatch between gauges lead to incomplete temperature compensation.

**Figure 8.**
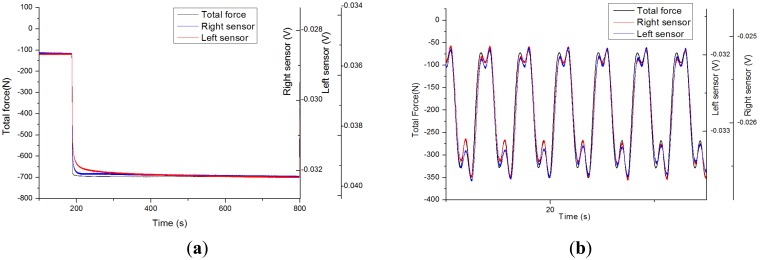
(**a**) Sensors response to force step between 110 N and 700 N on the tibial plate with 10° flexion angle. (**b**) Force pattern of walking with static 10° knee flexion angle.

The structure being made of composite and polymer materials, its viscoelastic behavior has an important impact on the performance of the sensor: testing its dynamic behavior is essential. The output voltage of the sensor under a force step actuation is represented in Figure 8a. Primary creep rate is high: a fast variation is observed upon force application. It is rapidly stabilized to a low steady-state or secondary creep rate: only a slow variation is observed when steady-state is reached. The observed low steady-state creep strain rate ensures a better stability and repeatability than typical behavior of sensors directly assembled to the polymer insert [12]. Creep, even though marginal, might still explain the slight hysteretic behavior observed in Figure 6 when ramping force up and down.

Realistic force patterns have been applied from recordings [18] on the device: such as slow walking M-shape patterns on an instrumented insert in a static knee flexion angle configuration, reported in Figure 8b. The shape described in [7] is observed here: force rises to the instant of contralateral toe off and contralateral heel strike and force diminishes to homolateral toe off and homolateral heel strike. In Figure 8b, the sensor output voltages follow the general shape of the applied force but for marginal divergence upon fast changes in forces due to some creep. If the sensitivity obtained in previous static calibration experiments is applied and electrical offset is set for obtaining the same average force level on the sensors, the obtained standard deviations are 26.9 N (0.99 Pearson r) and 18.2 N (0.99 Pearson r) for the right and left condyle sensors respectively. The sensor static calibration applies relatively closely with force patterns in the frequency range of this slow walking pattern.

Repeatability test was performed using a manually-operated knee rig previously described in [22]. For nine experiments over 2 h, applied force was manually ramped up and down at a rate of 200 N/min between 50 N and 900 N in a knee flexion angle of 15°. Time interval between each two consecutive experiments was 15 min. For the left condyle sensor, average sensitivity was 2.24 μV/N and the relative standard deviation, *i.e.*, normalized standard deviation by range, was 1.8%. For the right condyle sensor, average sensitivity was 3.55 μV/N and the relative standard deviation was 1.5%. The repeated calibrations show a good repeatability of the sensor's sensitivity. Offset standard deviation was 27.4 μV and 31.7 μV for the left and right sensors respectively. Such offset variations are due to changes in room temperature: effect which needs a compensation strategy for keeping a suitable resolution as explained in a later section.

## 4. Discussion

The device presented and characterized here paves the way to create instrumented prosthetics with stacked PCB capsules incorporated in polymer inserts. This structure does not modify the implant function and mechanics much, yet it yields force measurements and contains sufficient space for integration of other type of sensors, and all needed electronics for signal conditioning and wireless communication. Sensitivities and dynamic behavior have been assessed and fulfilled the requirements for the specified applicative purposes: the short and long term measurements of forces in the condyles of total condylar prosthetic knees. Through a redesign, such a sealed capsule could be adapted to other prosthetic joints using UHMWPE inserts such as hip or shoulder prosthesis.

Steady-state creep has limited the performance of strain gauges directly assembled to the UHMWPE insert. The viscoelastic behavior of the harder FR4 support structure has reduced to a negligible amount the secondary creep instabilities: the interface onto which the gauges are glued has very different mechanical properties. The shearing creep component is dampened by the harder low creep material while the deflection of the thin structure upon force application yields enough strain. Even though steady-state creep is low, a primary creep behavior can be noticed in Figure 5 potentially limiting the device performances at high frequencies. The hysteresis observed in Figure 4 is another limiting factor probably due to the polymeric nature of the insert. Further study of the frequency response of the sensor would deepen our understanding of the behavior of the device.

The sensor is not solely sensitive to forces: the output signal also depends on temperature and flexion angle. The temperature impacts both the resistor values and the dilation of the proof body and the integrated dummy resistors only partially compensated the thermal dependence of the output signal. The temperature in a total condylar prosthetic knee has been reported to increase up to 7 °C [23] during extensive use. This range would lead to a maximal uncertainty of approximately 35N on the force measured by our sensor which is a strong reduction in resolution and a major limitation in the prescribed applications. The easy integration of a discrete temperature sensor in the main PCB would allow for compensating this drift: a resolution of 0.1 °C on temperature would lead to an error of approximately 0.5 N. The variation in angle modifies greatly the sensitivity and output signal of the sensor through a change in contact point between the femoral component and the UHMWPE insert. Knowing the angle and compensating for the associated signal output difference could be performed by the addition of a magnetic kinematic sensor composed of a magnet and anisotropic magnetoresistive sensors as described in [19]. This sensor would allow both rejecting the effect of flexion on force measurement and continuous monitoring the flexion angle while still being fully integrated in the implant.

The device presented here is not ready for *in-vivo* measurements: the gluing in the UHMPWE insert and the connector prevent it. The integration of the wireless communication electronic circuitry would render the connector unnecessary: those components are proposed to be integrated in the thicker PCB layer following an architecture described in [22]. The incorporation of the system in the polymer insert is to be performed during manufacturing and should not modify the external surface or mechanical behavior of the insert which need to be validated through fatigue testing in the simulator. Patient-specific implants are becoming reality [24–27] and layer-by-layer rapid prototyping method such as laser sintering [28] could allow incorporating the sensors in a custom made implant. Compression molding is common for UHMWPE insert manufacturing and over molding is an option for module incorporation in the insert.

## 5. Conclusion

In this work, a closed and waterproof structure allowing force sensing through a MEMS piezoresistive sensor is assembled in a UHMWPE prosthetic knee insert. The sensor is placed in a sealed sandwich structure that could in the future host all needed electronic circuitry for signal processing, remote powering and wireless data transmission. The test device exposes the capsule to the side, has no embedded electronics and uses a wired connection for signal acquisition. A suitable assembling method and the wireless communication are to be implemented for any implantation to be possible. The sensor was tested with different static and dynamic force applications to the level of 1.5 body weight. Linearity and sensitivity are satisfactory while viscoelastic behavior is not a major limitation of the device. The effect of both flexion angle and temperature are explored and characterized and compensation strategies are proposed.

The use of such strain sensors in the context of instrumented joint prosthesis yields important information on the mechanical behavior of the implant both during intraoperative and postoperative periods. Important assessment of the placement and adjustment of the insert such as soft tissue unbalance and potentially condylar congruence, prosthesis loosening or luxation can be performed. The signals also depend on the mechanical state of the insert such as breaking or wear leading to material and shape modification: an important information during prosthesis aging. The sensor allows gaining a deep insight in the behavior of the prosthesis and has the potential for being an interesting diagnostic tool.

## Figures and Tables

**Table 1.** Static calibration results. Root Mean Square Error (RMSE) and maximum error are defined as deviations from the linear fit functions.

**Condyle Sensor**	**Knee Flexion Angle (°)**	**Sensitivity (μV/N)**	**Adjusted-Rsquare**	**RMSE (N)**	**Maximum Error (N)**
Right	10	6.1	0.98	46	87
Left	10	6.5	0.99	35	83
Right	60	4.3	0.99	18	49
Left	60	5.3	0.98	28	64
